# Ratio fluorescence detection of tetracycline by a Eu^3+^/NH_2_-MIL-53(Al) composite†

**DOI:** 10.1039/d0ra09185e

**Published:** 2021-01-12

**Authors:** Jing Chen, Yali Xu, Shuying Li, Fanghong Xu, Qian Zhang

**Affiliations:** Key Lab of Bioelectrochemistry & Environmental Analysis of Gansu, College of Chemistry and Chemical Engineering, Northwest Normal University Lanzhou 730070 P. R. China jchen@nwnu.edu.cn +86-931-7971275

## Abstract

Tetracycline detection has been a great concern because of its overuse and difficulty in degrading. Here, a detection method with ratio fluorescence was developed by synthesizing Eu^3+^ doped nanocomposites with NH_2_-MIL-53(Al) nanosheets. After adding tetracycline, the fluorescence intensity at 616 nm characteristic emission peak of Eu^3+^ was sensitized by the antenna effect generated from coordinating Eu^3+^ with tetracycline, but the fluorescence of NH_2_-MIL-53(Al) at 433 nm was quenched by the fluorescence resonance energy transfer between the Eu^3+^-tetracycline composition and NH_2_-MIL-53(Al). Therefore, the efficient detection of tetracycline was achieved based on this change of ratio fluorescence signal. The experimental results show that Eu^3+^/NH_2_-MIL-53(Al) has excellent selectivity, a wider linear range and a lower detection limit for detecting tetracycline. This method can afford favorable ideas for developing advanced chemical and biological sensors.

## Abstract 2

Tetracycline detection has been a great concern because of its overuse and difficulty in degrading.

## Graphical abstract

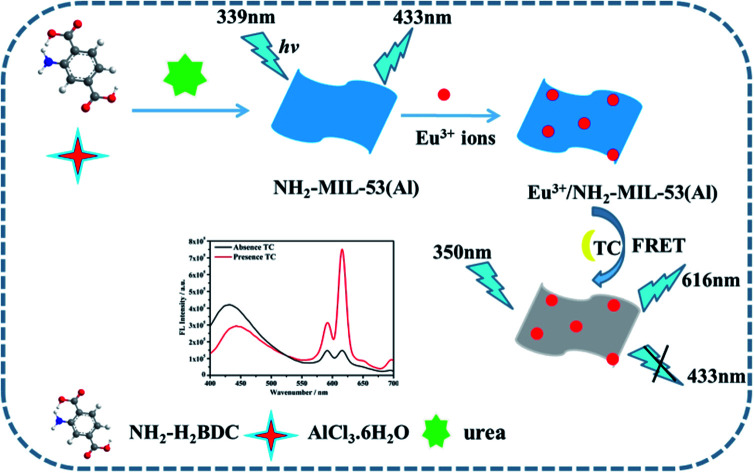

## 1. Introduction

Tetracycline (TC) has been used to treat bacterial infections due to its excellent antibacterial properties and good therapeutic effects.^1–3^ It is difficult to be degraded. Therefore, excessive use may cause it to accumulate in foods such as meat, eggs and milk, in water and soil, which seriously threatens human health.^4–6^ It is necessary to seek a highly sensitive and selective strategy to realize the detection of TC. At present, relatively mature detection methods include immunoassay, liquid chromatography-mass spectrometry, chemiluminescence, high performance liquid chromatography and so on.^7–10^ However, their applications are restricted owing to the methods are often time-consuming, requiring expensive equipment and complex sample preparation.^11^

Recently, novel fluorescence detection methods based on the design of highly effective fluorescence probes have caught widespread attention because of their obvious advantages, such as simple, sensitive, fast and easy operation with a lower cost. A variety of nanomaterial-based fluorescent probes have been reported for detecting TC, including water-soluble quantum dots (QD),^12^ metal nanoclusters (M NCs),^13^ and Ag nanoparticles (Ag NPs).^14^ These sensors usually detect the target object through the change of a single fluorescent signal. The accuracy of the detection results from these methods can be affected by some human and environmental factors.^15^ In order to effectively overcome these problems and achieve sensitive and accurate detection to a target, some ratiometric fluorescent probes have been proposed. For example, Li *et al.*^16^ designed a SiNPs-Eu sensor based on fluorescence resonance energy transfer for a ratio-type detection of TC. Recently, a new ratiometric fluorescence probe based on carbon dots was further developed for detecting TC.^12,17^

Liking metal–organic framework materials (MOFs), two-dimensional metal–organic framework nanosheets are coordinately assembled by metals and organic ligands. Compared with MOFs, MOFs nanosheets have many advantages, such as rich active sites, large specific surface area, high aspect ratio, adjustable porous structure and chemical composition, as well as ultra-thin thickness, which are widely used in the fields of catalysis, gas separation and chemical sensing.^18,19^ However, most MOFs are currently synthesized in organic solvent systems to ensure their topological structure, which leads to poor water stability and limits the application of water-soluble substances in aqueous systems.^20^ The introduction of NH_2_-MIL-53(Al) in water solvent through one-pot hydrothermal synthesis of –NH_2_ has the advantages of avoiding the complicated steps involved in post-modification and good water solubility. On the other hand, for the FL detection system, the introduction of –NH_2_ can improve the selectivity and sensitivity, thus significantly expanding the potential application of MOFs in FL analysis.^21–24^ The introduced –NH_2_ can react with TCs through hydrogen bond interaction, effectively speeding up the quenching of the fluorescence of NH_2_-MIL-53(Al). In addition, the MIL-type MOF composed of Al^3+^ and dicarboxylate ligand is stable to water and high temperature.^20,25–27^ Therefore, efficient sensors can be developed by using the advantages of the composite materials based on MOFs nanosheets.

**Scheme 1:**
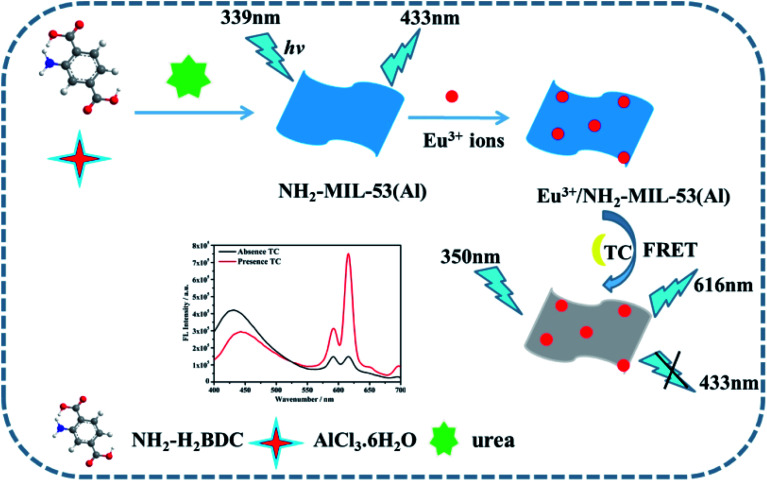
The synthesis of Eu^3+^/NH_2_-MIL-53(Al) composites and the mechanism for detecting TC.

In this paper, a novel ratio-type fluorescence sensor based on the composite material of MOFs nanosheets (Eu^3+^/NH_2_-MIL-53(Al)) was prepared and used for detecting tetracycline. First, the NH_2_-MIL-53(Al) nanosheets were prepared by hydrothermal method. The Eu^3+^/NH_2_-MIL-53(Al) nanocomposites were then obtained by doping Eu^3+^ ions into NH_2_-MIL-53(Al) nanosheets. Once tetracycline was added, Eu^3+^ acts as a response unit to coordinate with tetracycline to form Eu–TC complex, which sensitizes the characteristic emission peak of Eu^3+^ at 616 nm through the antenna effect. With the successive concentration increase of tetracycline, the characteristic the emission peak of Eu^3+^ at 616 nm increase continuously. Due to the overlap between the ultraviolet-visible absorption spectrum of Eu–TC complex and the fluorescence emission spectrum of NH_2_-MIL-53(Al), there might be a fluorescence resonance energy transfer effect between them, making the fluorescence intensity of NH_2_-MIL-53(Al) at 433 nm quenched, but the fluorescence intensity of Eu–TC complexes increases successively. Therefore, the highly sensitive detection of tetracycline can be achieved by using these ratio fluorescence signal change. The experimental results show that Eu^3+^/NH_2_-MIL-53(Al) has a relatively high selectivity, a wide linear range and a low detection limit for detecting tetracycline. The mechanism diagram of the ratio-type detection of tetracycline by Eu^3+^/NH_2_-MIL-53(Al) nanocomposite is shown in Scheme 1.

## 2. Experimental section

### 2.1 Chemicals and instruments

Europium oxide, tetracycline and hydrochloric acid are purchased from Aladdin. Tris(hydroxymethyl)aminomethane was obtained from Shanghai Zhongqin Chemical Reagent. Absolute ethanol was from Tianjin Anlong. All reagents are analytical pure grade. Europium chloride was prepared from europium oxide. The water used in the experiment was ultrapure water (18.2 MΩ cm). Fluorescence spectrum were recorded by a FluoroMax-4 fluorescence spectrophotometer (American FEI Corporation). Other instruments are the PHS-3B pH acidity meter (Shanghai Precision Instrument Science Co., Ltd.), the Nicolet Impact-400 Fourier infrared spectrometer (Shimadzu, Nicolet Corporation), the XRD-6000 X-powder diffractometer (Shanghai Rente Testing Instrument Co., Ltd.), the UV-1102 UV spectrophotometer (Shanghai Tianmei Scientific Instrument Company), the DZF-6020 vacuum drying oven (Shanghai Yiheng Technology Co. Ltd), the BSA224S electronic balance (Beijing Sedorius Scientific Instruments) and the PCD-2000 thermostatic blast dryer (Shanghai Langgan Shiyan Shebei Co. LTD.).

### 2.2 Preparation of NH_2_-MIL-53(Al) nanosheets

NH_2_-MIL-53(Al) nanosheets were prepared as follows.^28^ 3 mmol AlCl_3_·6H_2_O was dissolved into 15 mL ultrapure water. Then, 3 mmol NH_2_-H_2_BDC was added into the above solution under magnetic stirring. After stirring for 30 minutes, dropping 15 mL deionized water solution with 6 mmol urea into the above mixture, and stirring for 30 minutes. Furthermore, the prepared mixture was placed in a 70 mL autoclave and reacted in an oven at 150 °C for 5 hours. After cooling to the room temperature, the mixture was centrifuged at 8000 rpm and washed several times with ultrapure water. Then, the product was dispersed in 20 mL of DMF and 20 mL of methanol, and respectively stirred at room temperature for 1 day. Finally, the solvent was removed by centrifugation, and the desired product was obtained by drying.

### 2.3 Preparation of Eu^3+^/NH_2_-MIL-53(Al)

By referring the reported method and making slight modification, Eu^3+^/NH_2_-MIL-53(Al) composite material was prepared by chemical doping method.^29^ After immersing 50 mg NH_2_-MIL-53(Al) nanosheets into an ethanol solution containing EuCl_3_·6H_2_O (5 mL, 1 mmol) for 2 days, the undoped europium ions was removed by centrifuging and washing with ethanol solution. Then, the Eu^3+^/NH_2_-MIL-53(Al) nanocomposite was obtained by drying above remained solution at 60 °C.

### 2.4 Detection TC by NH_2_-MIL-53(Al) nanosheet

At room temperature, the specific process the fluorescence detection of tetracycline (pH = 5, 0.1 M) in Tris–HCl buffer solution was as follows. 2 mL Tris–HCl buffer solution (0.1 M, pH = 5) was added into 100 μL (0.005 mg mL^−1^) NH_2_-MIL-53(Al) aqueous solution. Then, the TC solutions with different concentrations were added into the above solution. Finally, the fluorescence intensity of NH_2_-MIL-53(Al) nanosheets was measured and observed at an excitation wavelength of 339 nm.

### 2.5 Eu^3+^/NH_2_-MIL-53(Al) nanocomposite testing TC

The specific process of fluorescence detection of tetracycline (pH = 9, 0.1 M) in Tris–HCl buffer solution at room temperature was as follows. 2 mL Tris–HCl buffer solution (0.1 M, pH = 9) was added into 100 μL (0.005 mg mL^−1^) Eu^3+^/NH_2_-MIL-53(Al) aqueous solution. Then, the TC solutions with different concentrations were added into the above solution, and reacting for 1 minute at room temperature. Finally, the change of the fluorescence signal of Eu^3+^/NH_2_-MIL-53(Al) nanocomposite was measured at the excitation wavelength of 339 nm.

## 3. Results and discussion

### 3.1 TEM image of NH_2_-MIL-53(Al)

**Fig. 1:**
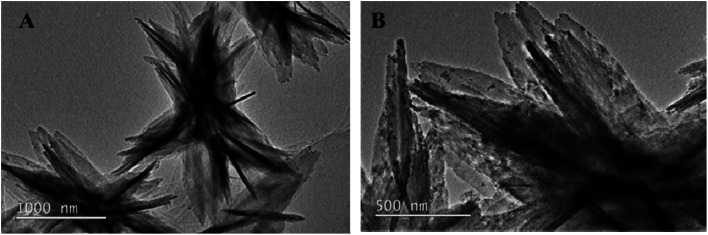
TEM image of NH_2_-MIL-53(Al).

The NH_2_-MIL-53(Al) nanosheets were characterized by TEM, PXRD, FT-IR and fluorescence spectroscopy. As shown in Fig. 1A and B, NH_2_-MIL-53(Al) is successfully prepared, and we can see the morphology of 2D nanosheets.

### 3.2 Powder X-ray diffraction (PXRD) and Fourier infrared spectroscopy (FT-IR) characteristics of Eu^3+^/NH_2_-MIL-53(Al)

**Fig. 2:**
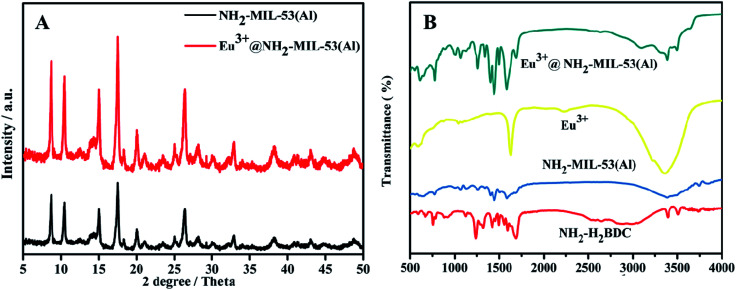
(A) PXRD diagram of NH_2_-MIL-53(Al) (black line) and Eu^3+^/NH_2_-MIL-53(Al) (red line). (B) FT-IR image of NH_2_–H_2_BDC (red line), NH_2_-MIL-53(Al) (blue line), Eu^3+^ (yellow line) and Eu^3+^/NH_2_-MIL-53(Al) (green line).

The X-ray diffraction (PXRD) Characteristics of NH_2_-MIL-53(Al)^28^ (black line) and Eu^3+^/NH_2_-MIL-53(Al) (red line) nanocomposites are shown in Fig. 2A. It is obvious that the crystal structure of NH_2_-MIL-53(Al) nanosheets is not changed after doping with Eu^3+^. In the Fourier infrared spectrum (FT-IR) of the nanocomposites (Fig. 2B), NH_2_-MIL-53(Al) nanosheets (blue line) is successfully prepared by comparing with the organic ligand NH_2_-H_2_BDC (red line). The slight shift of the peak in the Eu^3+^/NH_2_-MIL-53(Al) nanocomposite (green line) indicates that Eu^3+^ is doped into the NH_2_-MIL-53(Al) nanosheet.^29^

### 3.3 BET characterization of NH_2_-MIL-53(Al) and Eu^3+^/NH_2_-MIL-53(Al)

**Fig. 3:**
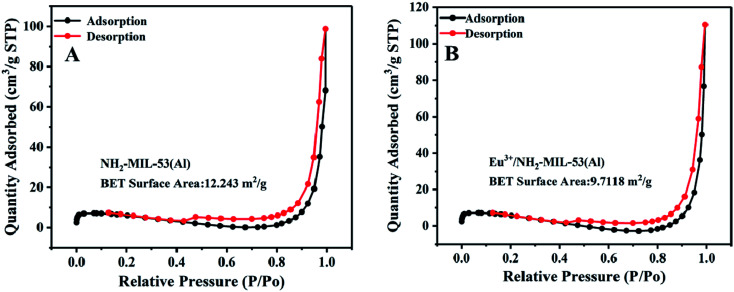
BET characterization of NH_2_-MIL-53(Al) (A)and Eu^3+^/NH_2_-MIL-53(Al) (B).

In order to further characterize the composite material, we use BET characterization, and the result is shown in Fig. 3.

### 3.4 Characterization of the ultraviolet and fluorescence spectra of the material Eu^3+^/NH_2_-MIL-53(Al)

**Fig. 4:**
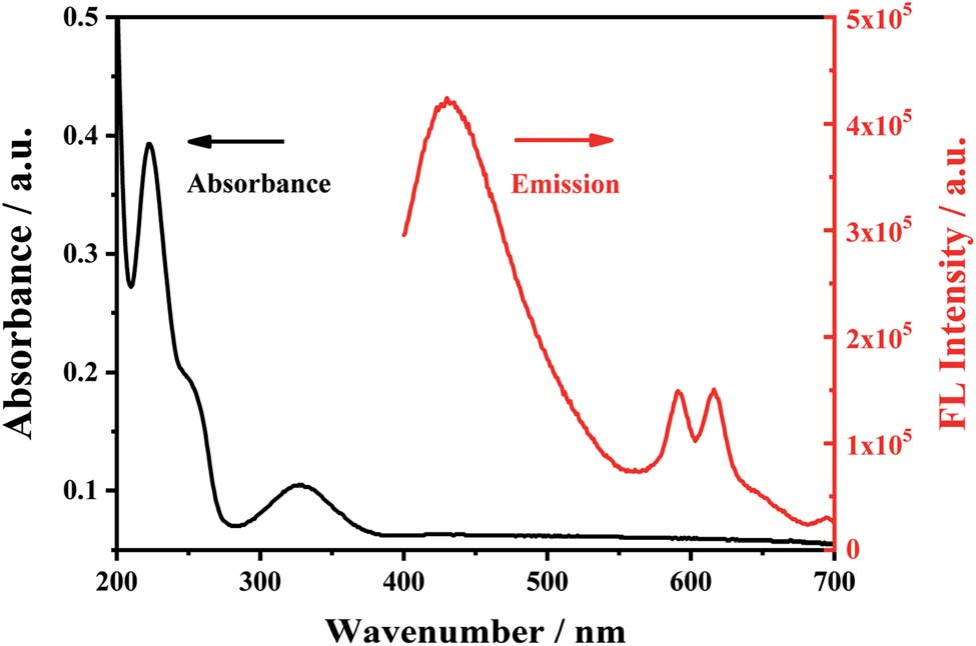
The UV-vis absorption spectra (black line) and fluorescence emission spectra of Eu^3+^/NH_2_-MIL-53(Al).

In the ultraviolet-visible absorption spectrum and fluorescence (FL) emission spectrum of Eu^3+^/NH_2_-MIL-53(Al) composites (Fig. 4), the Eu^3+^/NH_2_-MIL-53(Al) composite material has an absorption peak at 330 nm, and has the characteristic fluorescence emission peaks of NH_2_-MIL-53(Al) nanosheets at 433 nm and Eu^3+^ at 616 nm, indicating the Eu^3+^/NH_2_-MIL-53(Al) composites are prepared.

### 3.5 Condition optimization of the TC detection by NH_2_-MIL-53(Al) nanosheets

**Fig. 5:**
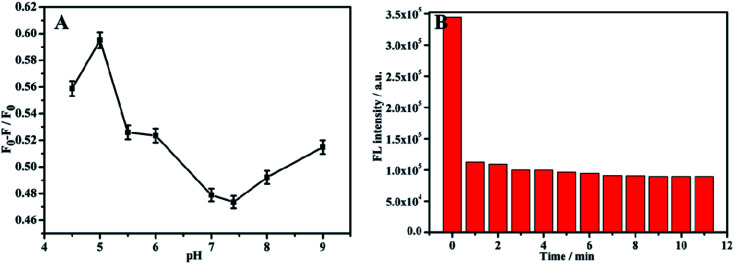
(A) Effect of different pH on TC detection of NH_2_-MIL-53(Al), (B) response time of NH_2_-MIL-53(Al) to TC.

Before using composite materials to detect TC, a single material NH_2_-MIL-53(Al) nanosheets was used to perform the fluorescent detection on TC. In order to improve the accuracy of the detection results, the pH and response time during the experiment were optimized. The fluorescence intensity changed with the change of the pH value within the range of 4–9, and had the most response when pH = 5 (Fig. 5A). So, pH = 5 was selected as the optimal pH value. During the experiment, 20 μL TC (10^−3^ M) was added dropwise (Fig. 5B). Once TC was added, the fluorescence intensity of the nanosheets decreased rapidly, and did not change apparently with the increase of time. It is obvious that the fluorescence intensity of NH_2_-MIL-53(Al) can be barely impacted by the response time.

### 3.6 Fluorescence detection of TC by NH_2_-MIL-53(Al)

**Fig. 6:**
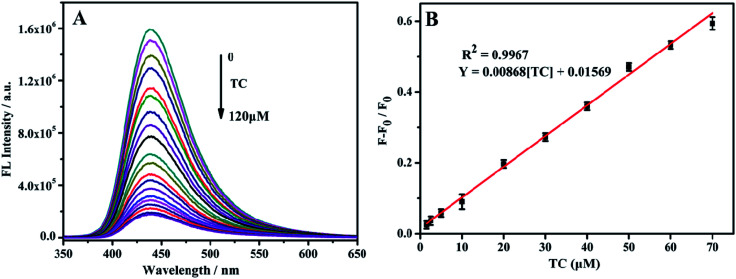
(A) The fluorescence emission spectra of NH_2_-MIL-53(Al) with the increase of TC concentration; (B) NH_2_-MIL-53(Al) nanosheets linear relationship between changes in fluorescence intensity and TC concentrations.

TC was detected with the above-mentioned optimal conditions. With the TC concentration increase from 0–120 μM, the fluorescence intensity at 433 nm gradually decreases (Fig. 6A). Within the range of 1.5–70 μM TC concentration, the change in fluorescence intensity ((*F*_0_ − *F*)/*F*_0_) shows a good linear relationship with the TC concentration (Fig. 6B). The linear regression equation is (*F*_0_ − *F*)/*F*_0_ = 0.04409 [TC] + 0.01748, and the calculated minimum detection limit (LOD) is 0.92 μM.

### 3.7 Fluorescence detection of TC by Eu^3+^/NH_2_-MIL-53(Al)

**Fig. 7:**
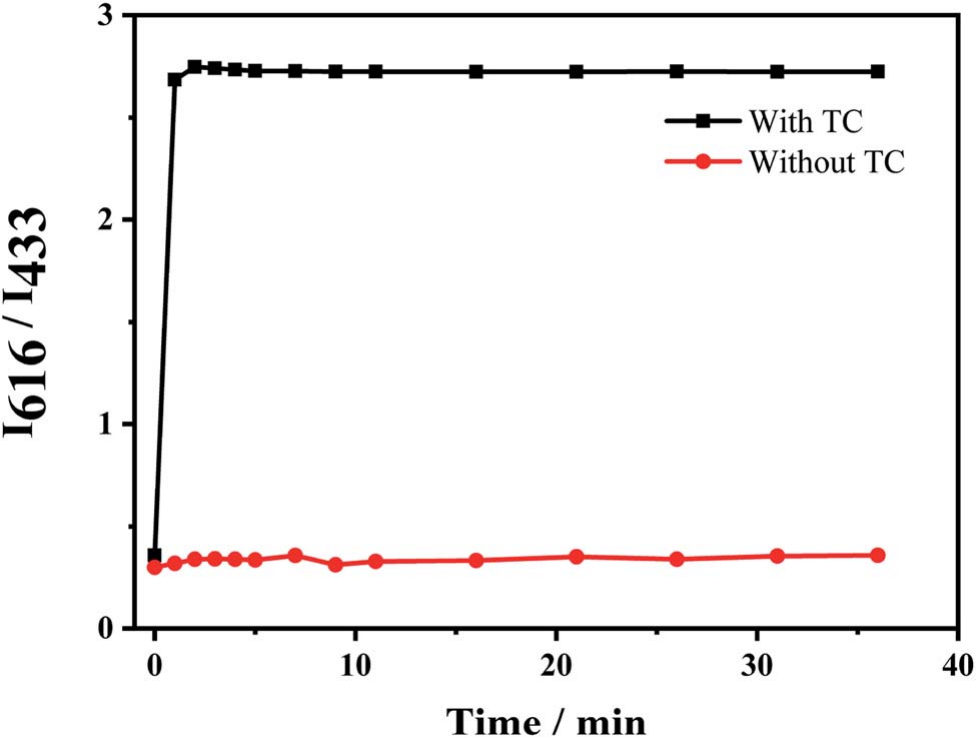
Response time of Eu^3+^/NH_2_-MIL-53(Al) to TC fluorescence detection.

The response time of the fluorescent probe Eu^3+^/NH_2_-MIL-53(Al) for detecting tetracycline was studied (Fig. 7). The response time between the fluorescent signal of Eu^3+^/NH_2_-MIL-53(Al) composite and TC is very fast, which can be completed within 1 min. The fluorescence intensity ratio (*I*_616_/*I*_433_) basically unchanges with the further increase of the response time, indicating that the probe has a better response and stability for detecting TC.

**Fig. 8:**
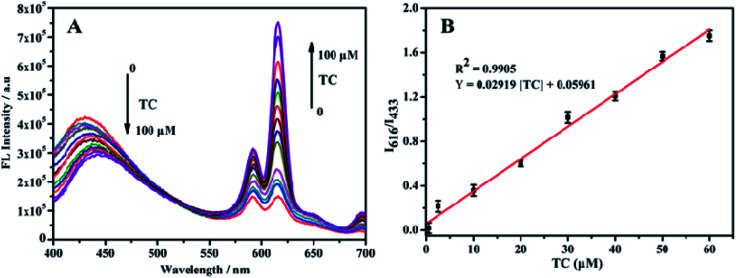
(A) The change in the ratio fluorescence (*I*_616_/*I*_433_) intensity of Eu^3+^/NH_2_-MIL-53(Al) after adding different concentrations of TC; (B) the linear relationship between TC concentrations and fluorescence intensity ratio (*I*_616_/*I*_433_).

Fig. 8A shows the response of TC solutions with different concentrations to the fluorescence intensity ratio (*I*_616_/*I*_433_) of Eu^3+^/NH_2_-MIL-53(Al) composite material. With the increase of the TC concentration, the fluorescence intensity of NH_2_-MIL-53(Al) at 433 nm is getting smaller and smaller, but the fluorescence intensity of Eu^3+^ is increasing at 616 nm. Therefore, the Eu^3+^/NH_2_-MIL-53(Al) composite material can be used as a ratiometric fluorescent probe for detecting TC. Fig. 7B is the relationship between different TC concentrations and the fluorescence intensity ratio (*I*_616_/*I*_433_). When the TC concentration changes from 0.5 to 60 μM, the concentration of TC and *I*_616_/*I*_433_ shows a good linear relationship. The linear equation is *I*_616_/*I*_433_ = 0.02919 [TC] + 0.05961 with a correlation coefficient *R*^2^ = 0.9905. The minimum detection limit of the composite material for TC is 0.16 μM, compared to the detection of TC by NH_2_-MIL-53(Al) nanosheets, the ratiometric fluorescent probes have a distinct lower detection limit.

### 3.8 The selectivity of the TC detection by Eu^3+^/NH_2_-MIL-53(Al)

**Fig. 9:**
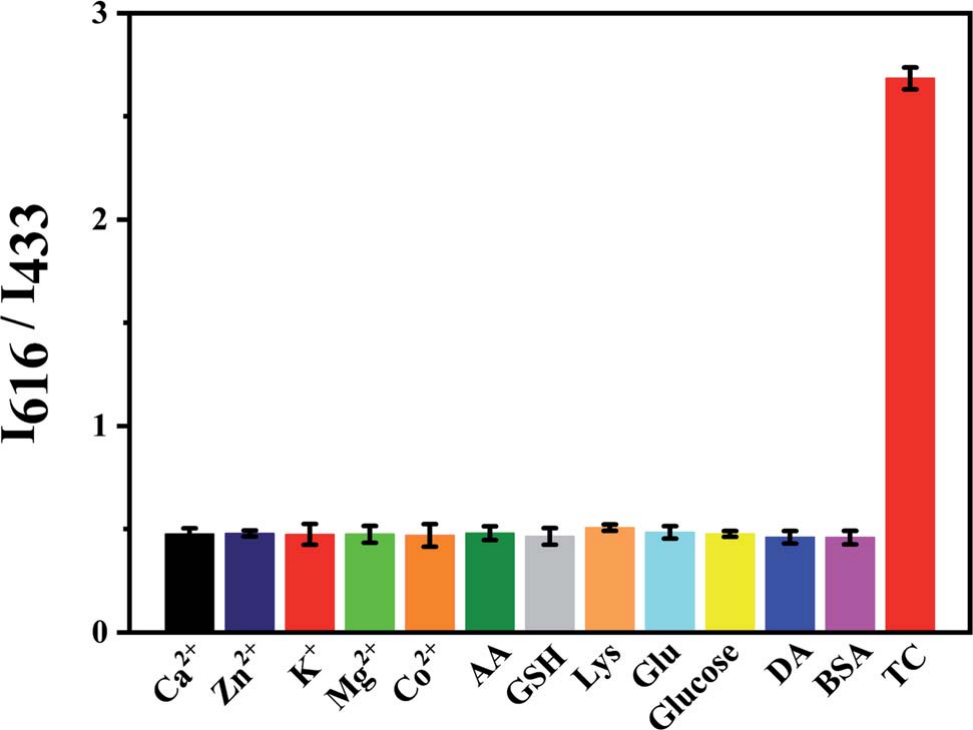
Selectivity detect of Eu^3+^/NH_2_-MIL-53(Al) for TC.

The effect of some interferences like Co^2+^, Ca^2+^, Mg^2+^, Zn^2+^, K^+^, ascorbic acid (AA), cysteine (Lys) and glutathione (GSH) to the fluorescence intensity ratio (*I*_616_/*I*_433_) of Eu^3+^/NH_2_-MIL-53(Al) composite material was studied (Fig. 9). The results show that the fluorescence intensity ratio is obviously changed by adding TC, while other interferences have little effect on the fluorescence intensity ratio, indicating that the nanocomposite can achieve TC detection.

### 3.9 Detection of TC in water samples

In order to further evaluate the anti-interference ability of fluorescent probes and the feasibility of this dual emission ratio fluorescent probe for rapid and ultra-sensitive detection of TC in practical applications, we performed fluorescence spectroscopy on actual water samples (tap water) test. As shown in the ESI Table 2,† the fluorescence intensity ratio of tap water and deionized water solution gradually increases with the increase of TC concentration. In addition, the ratio of the fluorescence intensity of the probes in the two water samples was approximately the same, and no significant difference was observed. The fluorescence intensity ratio has a linear relationship with the TC concentration and a good recovery rate of standard addition is obtained. The results show that the detection system has the same results as the laboratory conditions of deionized water, which can effectively eliminate the interference of coexisting substances, which proves that the probe has excellent selectivity. At the same time, it proved the accuracy and reliability of the probe in measuring TC in environmental samples.

### 3.10 The mechanism verification of TC detection by Eu^3+^/NH_2_-MIL-53(Al)

**Fig. 10:**
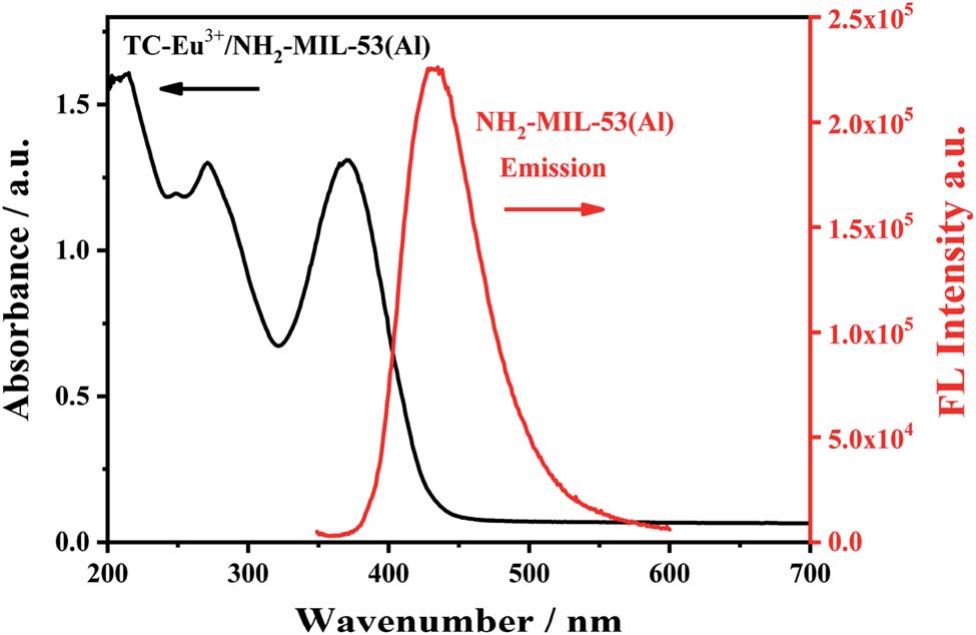
The UV-vis absorption spectrum (black line) of TC–Eu^3+^/NH_2_-MIL-53(Al) and the fluorescence emission spectrum of NH_2_-MIL-53(Al).

In order to study the proposed detection mechanism, the characterization of ultraviolet-visible absorption spectrum and fluorescence spectrum was carried out. There is a spectral overlap between the ultraviolet-visible absorption spectrum of the complex TC–Eu^3+^/NH_2_-MIL-53(Al) and the fluorescence spectrum of NH_2_-MIL-53(Al) (Fig. 10), indicating that there may have a fluorescence resonance energy transfer effect between them. As an energy donor, the fluorescence intensity of NH_2_-MIL-53(Al) is quenched, while the fluorescence intensity of the TC–Eu^3+^/NH_2_-MIL-53(Al) complex as an energy acceptor is enhanced. Thereby, detecting tetracycline can be achieved by changing the ratio of fluorescence signals.

## 4. Conclusion

In summary, a ratio-based fluorescence detection method is proposed for detecting TC by preparing Eu^3+^/NH_2_-MIL-53(Al) nanocomposite. When TC is added, Eu^3+^ acts as a response unit to coordinate with tetracycline to form a complex, thereby sensitizing the characteristic emission peak of Eu^3+^ at 616 nm through the antenna effect. Due to the spectral overlap between the ultraviolet-visible absorption spectrum of the formed complex and the fluorescence spectrum of NH_2_-MIL-53(Al), indicating that there may be a fluorescence resonance energy transfer effect between them, so the fluorescence intensity of NH_2_-MIL-53(Al) at 433 nm is quenched. With the increase of TC concentration, the fluorescence at 433 nm decreases continuously, and the fluorescence at 616 nm increases. So, the purpose of detecting tetracycline can be achieved by changing the ratio of fluorescence signals. The linear range of this ratio fluorescent probe for detecting tetracycline is 0.5–60 μM, the minimum detection limit is 0.16 μM. The method provides a novel idea for detecting tetracycline with excellent selectivity.

## Conflicts of interest

There are no conflicts to declare.

## Supplementary Material

RA-011-D0RA09185E-s001
