# Supplementary material for: Ratio fluorescence detection of tetracycline by a Eu3+/NH2-MIL-53(Al) composite
Source: RSC Adv. 2021 Jan 12;11(4):2397–404. doi: 10.1039/d0ra09185e (PMC8693705; doi:10.1039/d0ra09185e)
Supplement: RA-011-D0RA09185E-s001 [file RA-011-D0RA09185E-s001.pdf]

Electronic Supplementary Material (ESI) for RSC Advances.
This journal is © The Royal Society of Chemistry 2020

Ratio Fluorescence Detection of Tetracycline by Eu^{3+}/NH_2-MIL-53(Al) composite

Jing Chen*, Yali Xu, Shuying Li, Fanghong Xu, Qian Zhang

Key Lab of Bioelectrochemistry & Environmental Analysis of Gansu, College of Chemistry and Chemical Engineering, Northwest Normal University, Lanzhou 730070, China

Corresponding address:

Key Lab of Bioelectrochemistry & Environmental Analysis of Gansu,

College of Chemistry and Chemical Engineering,

Northwest Normal University,

Lanzhou, 730070, P. R. China

Tel: +86-931-7971275

E-mail: jchen@nwnu.edu.cn

* Corresponding author.

Table 1 Comparison of This Method with Reported Tetracycline Sensors.

Methods	Materials	Linear range	LOD	Ref.
LC-MS/MS	-	25-200 μg /kg	2.22-3.59 μg/kg	1
CE	-	25-250 μg/L	2-9 μg/L	2
Fluorometry	FeOx@SiO_2 -FMIPs	0.2-6 μM	117 nM	3
Fluorometry	CDs	2-150 μM	520 nM	4
Fluorometry	Eu-EDTA+surfactant	0.2-5 μM	0.2 μM	5
Fluorometry	NH_2-MIL-53(Al)	1.5-70 μM	0.92 μM	This work
Fluorometry	Eu^{3+}/NH_2-MIL-53(Al)	0.5-60 μM	0.16 μM	This work

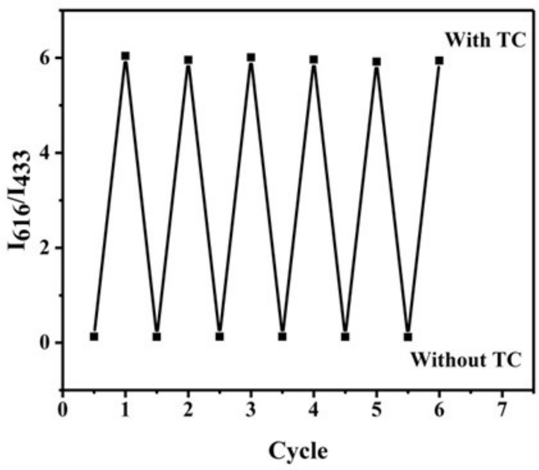

Fig. S1 The fluorescence reversibility of the probe in aqueous solution

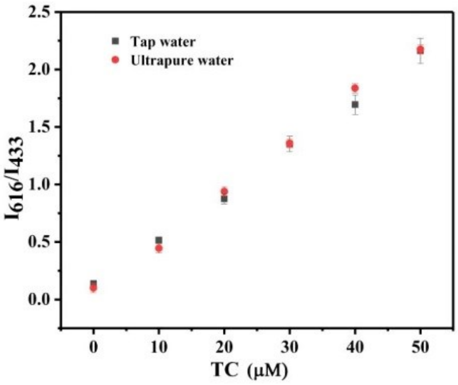

Fig. S2 The linear relationship between the TC concentration of different concentrations in the actual water sample and the ratio of the fluorescence intensity of the probe

Table 2 The Results for the Determination of TC in Real Samples.

Sample	Added (μM)	Founded (μM)	Recovry (n=3,%)	RSD (n=3,%)
	20	20.6526	103.26	5.9966
Tap water	30	30.4321	101.44	1.5346
	40	38.0127	95.03	2.4287

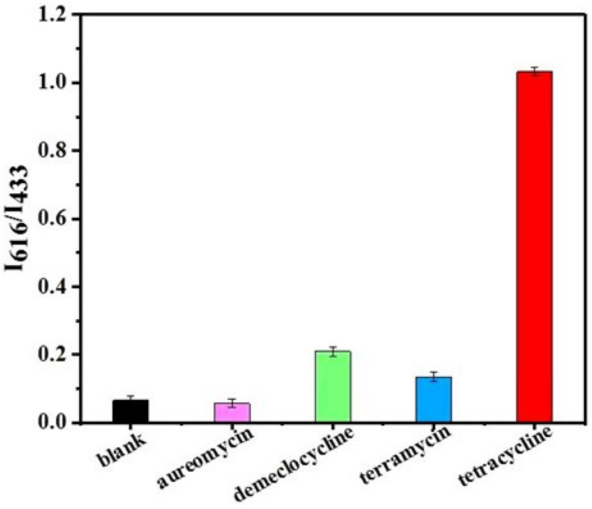

Fig. S3 Selectivity of fluorescent probes to aureomycin, terramycin, tetracyclne, demeclocycline at room temperature

[1] S. O. S. Mookantsa, S. Dube, M. M. Nindi, Development and application of a dispersive liquid-liquid microextraction method for the determination of tetracyclines in beef by liquid chromatography mass spectrometry. Talanta 148 (2016) 321-328.

[2] Rodríguez Ávila, José Antonio. Magnetic solid phase extraction based on phenyl silica adsorbent for the determination of tetracyclines in milk samples by capillary electrophoresis. J. Chromatogr. A 1218 (2011) 2196-2202.

[3] J. Ashley, X. T. Feng, Y. A Sun, multifunctional molecularly imprinted polymer-based biosensor for direct detection of doxycycline in food samples. Talanta 182 (2018) 49-54.

[4] M. Lin,; H. Y. Zou,; T.Yang,et al. An inner filter effect based sensor of tetracycline hydrochloride as developed by loading photoluminescent carbon nanodots in the electrospun nanofibers. Nanoscale 8 (2016) 2999-3007.

[5] J. Zhang, B. Zhang, Y. Wu, et al. Fast determination of the tetracyclines in milk samples by the aptamer biosensor. Analyst 135(10) (2010) 2706-2710.
